# Crystal Structures of Botulinum Neurotoxin Subtypes A4 and A5 Cell Binding Domains in Complex with Receptor Ganglioside

**DOI:** 10.3390/toxins14020129

**Published:** 2022-02-08

**Authors:** Kyle S. Gregory, Otsile O. Mojanaga, Sai Man Liu, K. Ravi Acharya

**Affiliations:** 1 Department of Biology and Biochemistry, University of Bath, Claverton Down, Bath BA2 7AY, UK; kg540@bath.ac.uk (K.S.G.); oom21@bath.ac.uk (O.O.M.); 2 Protein Sciences Department, Ipsen Bioinnovation Limited, 102 Park Drive, Milton Park, Abingdon OX14 4RY, UK; sai.man.liu@ipsen.com

**Keywords:** botulinum neurotoxin, crystal structure, cell binding domain, subtypes A4 and A5, ganglioside binding

## Abstract

Botulinum neurotoxins (BoNT) cause the potentially fatal neuroparalytic disease botulism that arises due to proteolysis of a SNARE protein. Each BoNT is comprised of three domains: a cell binding domain (H_C_), a translocation domain (H_N_), and a catalytic (Zn^2+^ endopeptidase) domain (LC). The H_C_ is responsible for neuronal specificity by targeting both a protein and ganglioside receptor at the neuromuscular junction. Although highly toxic, some BoNTs are commercially available as therapeutics for the treatment of a range of neuromuscular conditions. Here we present the crystal structures of two BoNT cell binding domains, H_C_/A4 and H_C_/A5, in a complex with the oligosaccharide of ganglioside, GD1a and GM1b, respectively. These structures, along with a detailed comparison with the previously reported apo-structures, reveal the conformational changes that occur upon ganglioside binding and the interactions involved.

## 1. Introduction

Botulinum neurotoxin serotype A (BoNT/A) is produced by anaerobic spore forming bacteria, *Clostridium botulinum*, and, along with other serotypes, is responsible for the disease *botulism*—a neuromuscular condition that causes flaccid paralysis and can lead to death by asphyxiation if left untreated [1]. The exquisite toxicity of BoNT/A makes it one of the deadliest agents known to humankind [2]; however, at miniscule doses, they can be used as a therapeutic to treat a range of diseases associated with hyper-muscular and -glandular activity [3]. The toxin is post-translationally cleaved to form an active di-chain, comprised of a 50 kDa light chain (LC) and a 100 kDa heavy chain (HC) linked by a disulphide bond. The HC can be further divided into an N-terminal translocation domain (H_N_) and a C-terminal cell binding domain (H_C_) [4]. The mechanism of intoxication involves three general steps [5]: highly specific targeting to the neuromuscular junction by dual-receptor recognition of both a protein and ganglioside receptor by the H_C_ domain, resulting in endocytic internalisation into an endosome [6]; pH-mediated conformational change of the H_N_ domain that translocates the LC into the cytosol [7,8,9]; and a Zn^2+^-dependent endopeptidase cleavage of a soluble *N*-ethylmaleimide-sensitive factor attachment protein receptor (SNARE) protein by the LC [10]. This cleavage prevents vesicular fusion to the cell membrane, halting the release of presynaptic acetylcholine, and the progression of synaptic signalling at the neuromuscular junction [11].

There are many different types of BoNT and BoNT-like molecules that are categorised by sequence similarity, serological activity, and/or host source. BoNTs produced by Clostridia are categorised into serotypes /A to /G, and /X, whereas the BoNT-like molecules by non-Clostridia include BoNT/Wo (*Weissella oryzae*) [12], BoNT/En (*Enterococcus faecium*) [13], and PMP1 (*Paraclostridium bifermentans*) [14]. Some serotypes exist naturally as mosaics (e.g., BoNT/CD, BoNT/DC, and BoNT/FA), whereas other serotypes are divided into subtypes (e.g., BoNT/A1-/A8, /B1-/B8, /E1-/E12, /F1-/F9) due to subtle variations in amino acid sequence [15,16]. Although these subtypes arise due to only minor changes in their amino acid sequence, the toxicity of subtypes has been shown to vary significantly [17,18,19,20]. All serotypes require recognition of both a protein (Synaptic vesicle protein 2 in BoNT/A) and ganglioside receptor to initiate endocytosis, except for BoNT/C which binds to two gangliosides. Gangliosides are glycosphingolipids that are often involved in cellular-signalling pathways and are comprised of a membrane anchored hydrophilic lipid tail, and an extracellular oligosaccharide moiety [21]. Previous studies have reported the structures of the binding domain of BoNT/A1 (H_C_/A1) and BoNT/A3 (H_C_/A3) in complex with the receptor ganglioside GD1a [22,23], detailing the interactions that occur between the two. These structures reveal that the ganglioside binding site (GBS) is formed by a β-hairpin and loop in the C-terminal subdomain of H_C_ (H_CC_).

We have previously reported the crystal structures of H_C_/A4 [24] and H_C_/A5 [25], and now present the crystal structures of H_C_/A4 in complex with GD1a, and H_C_/A5 in complex with GM1b, and highlight the interactions and structural changes that occur upon ganglioside binding. The structural information revealed in this report may aid in the development of future BoNT therapeutics.

## 2. Results and Discussion

### 2.1. Structure of H_C_/A4 in Complex with GD1a Oligosaccharide

**Figure 1:**
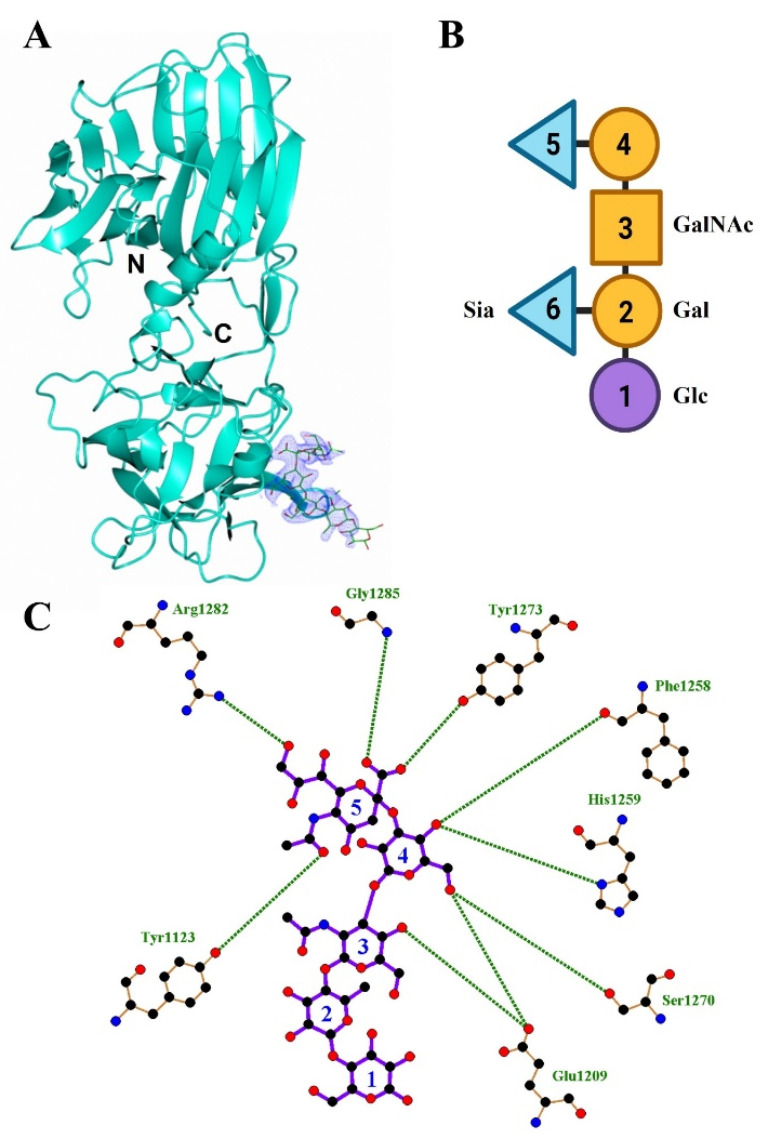
Structure of H_C_/A4:GD1a. (**A**) Crystal structure of H_C_/A4 in complex with GD1a oligosaccharide, the electron density map of GD1a (2F_O_-F_C_) is contoured to 1 σ; (**B**) schematic diagram of GD1a ganglioside; (**C**) LigPlot of GD1a and H_C_/A4 hydrogen bonding interactions.

The structure of the H_C_/A4:GD1a complex was solved to 2.3 Å by molecular replacement using the unbound H_C_/A4 structure (PDB: 6F0P) [24] as a search model. Two molecules (designated A and B) were present in the asymmetric unit (ASU) (Table 1). The overall quality of the electron density map was good with better density for molecule A (residues 994-999, 1029-1032,1047-1053, 1172-1174, and 1232-1239 could not be modelled for molecule B). Consequently, all subsequent analyses below involved molecule A. An initial inspection of the map revealed large positive electron density at the expected GBS, which indicated that GD1a had bound. Monosaccharides Sia^5^-Gal^2^ could be modelled with no ambiguity into the electron density (Figure 1A) and Glc^1^ partially, but there was insufficient electron density to model Sia^6^. A total of nine hydrogen bonding interactions were present between H_C_/A4 and GD1a (Figure 1B) (Table 2)—there was clear electron density for the two terminal nitrogen atoms of Arg 1282 which interact with Sia^5^ and Gln 1276.

**Figure 2:**
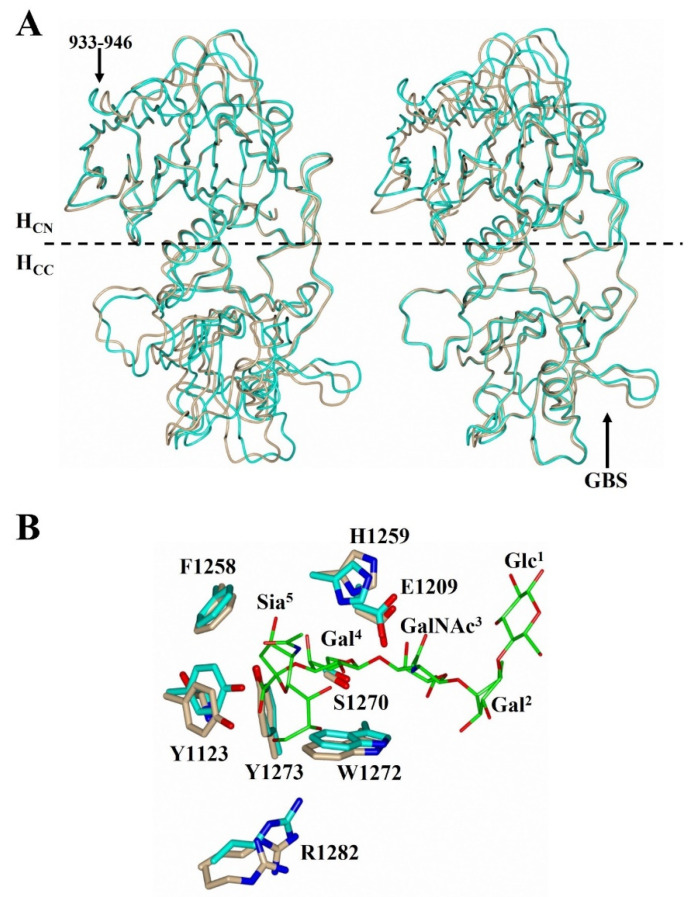
Comparison of H_C_/A4:GD1a with H_C_/A4. (**A**) Superimposition of the H_CN_ (**left**) and H_CC_ (**right**) domains of H_C_/A4:GD1a (cyan) and H_C_/A4 (burlywood; PDB: 6F0P [22]) shows a hinge movement between the subdomains; (**B**) Comparison of H_C_/A4:GD1a and H_C_/A4 at the ganglioside binding site.

The crystal packing of H_C_/A4 changes significantly upon binding of GD1a as evidenced by the change in both unit cell dimensions and space group. Although there is minimal overall conformational change between H_C_/A4:GD1a with H_C_/A4 alone (RMSD of 0.88 Å for all Cα atoms); there is a noticeable change in the relative position of the H_CN_ and H_CC_ subdomains when compared to the unbound structure (RMSD of 0.6 and 0.5 Å, respectively, for all Cα atoms after individual subdomain superimposition). Therefore, upon ganglioside binding, the two subdomains appear to rotate apart like an opening hinge (Figure 2A). There is also a noticeable conformational change to the loop spanning residues 933-946 within the H_CN_ subdomain (Figure 2A) that may be attributed to different crystal packing in the unbound structure.

Inspection of the H_C_/A4:GD1a GBS residues revealed changes in the relative position of the side chains compared to H_C_/A4 alone; most notably Arg 1282 (which adopts two conformations in the unbound structure) and Tyr 1123. Upon GD1a binding, these residues shift to form a hydrogen bonding interaction with Sia^5^ (Figure 2B).

### 2.2. Structure of H_C_/A5 Co-Crystallised with GM1b Oligosaccharide

**Figure 3:**
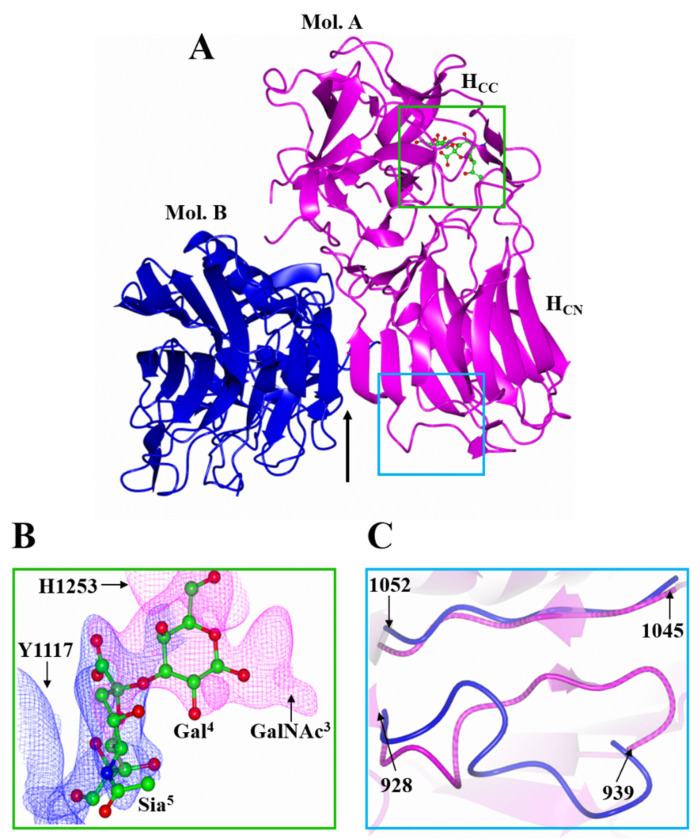
Crystal structure of H_C_/A5:GM1b. (**A**) The structure of H_C_/A5:GM1b was solved with two molecules in the ASU designated ‘A’ (magenta) and ‘B’ (blue). The arrow indicates the location of the crystallographic dimer interface; (**B**) Ganglioside binding site of molecule ‘A’. The polder maps for Gal^4^ (magenta) and Sia^5^ (blue) are contoured to 3 σ. His 1253 and Tyr 1117 have been omitted for clarity; (**C**) Superimposition of molecule ‘A’ and ‘B’ shows a change in conformation of the loop spanning residues 928-939.

Several attempts to crystallise H_C_/A5 with GD1a did not yield crystals for the complex. Consequently, a smaller ganglioside, GM1b, which is identical to GD1a in terms of the expected binding portion (Sia^5^-GalNAc^3^) but lacks only Sia^6^ (Figure 1B), was used for co-crystallisation with H_C_/A5. Crystals of H_C_/A5:GM1b diffracted to 2.4 Å, in space group P2_1_ (Table 1), and the structure was determined by molecular replacement with two molecules (designated A and B) in the ASU (Figure 3A). Molecule A generally showed clearer electron density throughout the structure compared to molecule B, especially the H…SxWY motif that is essential for ganglioside binding [26] which could not be modelled in molecule B. There are, however, three small loop regions in molecule A (1167-1169, 1226-1235, and 1271-1276) that showed insufficient density for modelling. It is possible that the latter loop (residues 1271-1276) is flexible due to its proximity to the GBS.

Both molecules A and B are conformationally very similar, with an RMSD of 0.68 Å for all Cα atoms. Residues 928-939, however, adopt alternative conformations—for molecule A they form a β-strand with residues 1047-1050 of the conserved jelly-roll fold, whereas for molecule B, they form an unstructured loop (Figure 3C). Inspection of the surrounding symmetry-related molecule suggests that this difference may be due to crystallographic packing.

**Figure 4:**
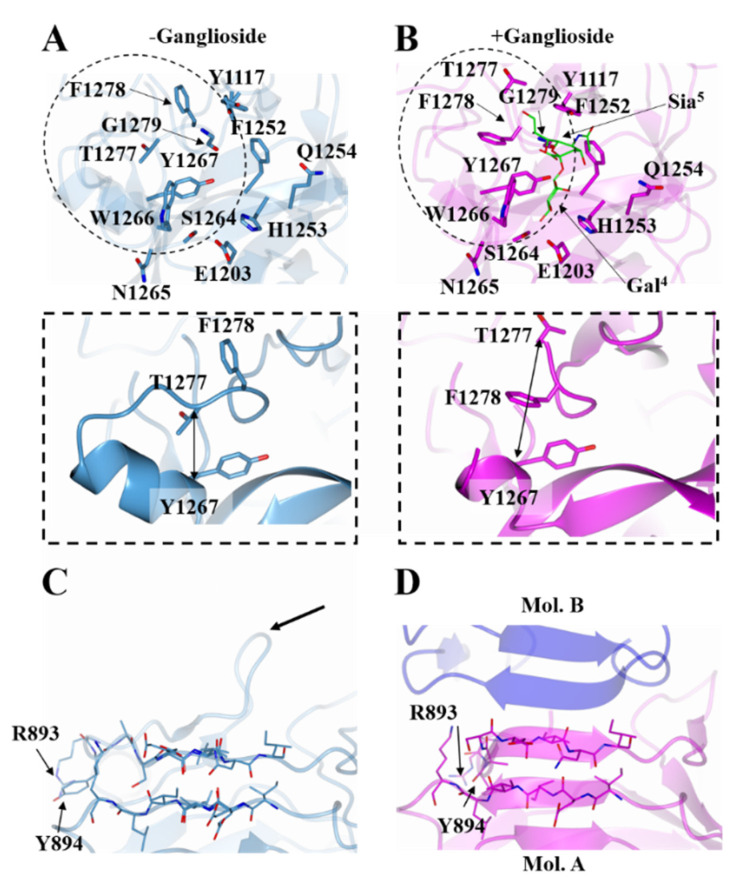
Comparison of H_C_/A5:GM1b with H_C_/A5. Structure of the GBS of H_C_/A5 with and without GM1b oligosaccharide (**B** and **A** (PDB: 6TWP [22]), respectively). The widening of the loop region spanning residues 1260-1280 is indicated by double-headed arrows (inset). Structure of H_C_/A5 N-terminus with and without GM1b oligosaccharide (**D** and **C**, respectively). The former shows the crystallographic dimer interface between molecule ‘A’ (magenta) and ‘B’ (blue). Arrow points to the location of loop 882-889.

The two molecules of the ASU form a dimer through an extended β-sheet interaction (Figure 3A, Arrow). For the interaction to occur the 882-889 loop, which extends beyond the β-sheet (Figure 4C, Arrow), has to move away to allow for the interface to form between molecule A and B. Although computational analyses [27] suggest this may also be due to crystallographic packing, the GBS in this crystal form has become accessible to ligand binding. For molecule A, some additional weak electron density was observed at the GBS that was not part of the protein. With the aid of polder maps [28] for His 1253 and Tyr 1117 [22,23,29], it was possible to model in sugars Gal^4^ and Sia^5^ (Figure 3B). Gal^4^ forms a total of four hydrogen bonds with residues Glu 1203, Phe 1252, His 1253, and Ser 1264, while Sia^5^ forms three hydrogen bonds with Tyr 1117, Tyr 1267, and Gly 1279 (Table 2). Further refinement of the Gal^4^ molecule with occupancies 0.6 and 1 generated average B-factors of 60.74 Å^2^ and 61.97 Å^2^, respectively, indicating that GM1b is bound at low occupancy.

The β-sheet arrangement between the two molecules of the ASU, shows a significant conformational change at the N-terminus of the H_C_/A5:GM1b structure when compared to H_C_/A5 alone (Figure 4C,D). Most prominently, the side chains of Arg 893 and Tyr 894 have rotated towards the main body of the protein structure upon GM1b binding, and there is a rotation in the protein backbone that results in a more compact structure. This closely resembles the full-length structure of BoNT/A1 (PDB:3BTA) in the absence of ganglioside.

Overall, the H_C_/A5:GM1b structure is very similar to the H_C_/A5 structure (PDB:6TWP), with RMSD values of 0.76 Å (for Cα atoms) for molecule A and 0.64 Å for molecule B (for Cα atoms). Considering that the residues of the GBS for molecule B could not be modelled, comparisons to the unbound H_C_/A5 structure will be made with molecule A. Most of the residues within the putative GBS show little to no conformational change, with the exception of Phe 1278 which has its side chain flipped towards the GBS (Figure 4A,B). This flip in residue positioning is accompanied by a change in the loop structure spanning residues 1260-1280, where there is an increase in the C^α^ distance of 4 Å between residues Tyr 1267 and Thr 1277 upon ganglioside binding (Figure 4A,B inset). This increase in C^α^ distance is indicative of the loop widening to accommodate the ganglioside.

### 2.3. Structural Variability of H_C_/A Subtypes at the Ganglioside Binding Site

**Figure 5:**
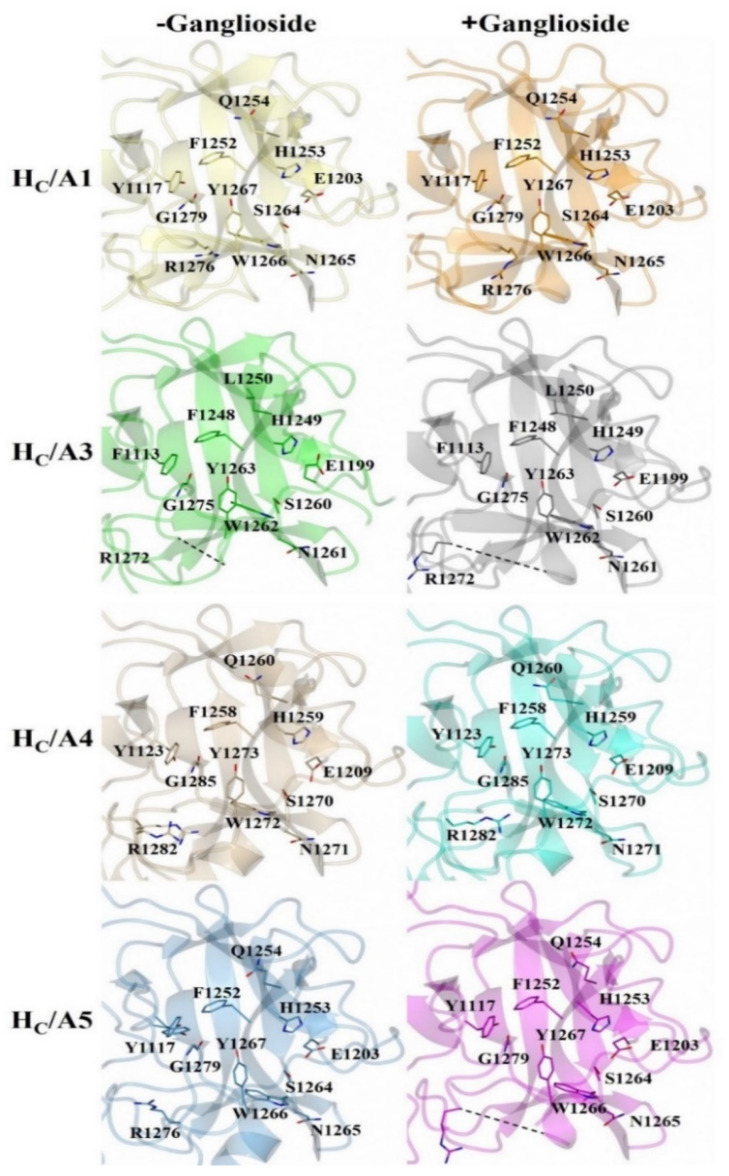
Structural comparison of the ganglioside binding site with and without oligosaccharide. H_C_/A1 (PDB: 2VUA [29]), H_C_/A1:GD1a (PDB: 5TPC [22]), H_C_/A3 (PDB: 6F0O [22]), H_C_/A3:GD1a (PDB: 6THY [22]), H_C_/A4 (PDB: 6F0P [24]), H_C_/A4:GD1a (this study), H_C_/A5 (PDB: 6TWP [24]), and H_C_/A5:GM1b (this study).

**Figure 6:**
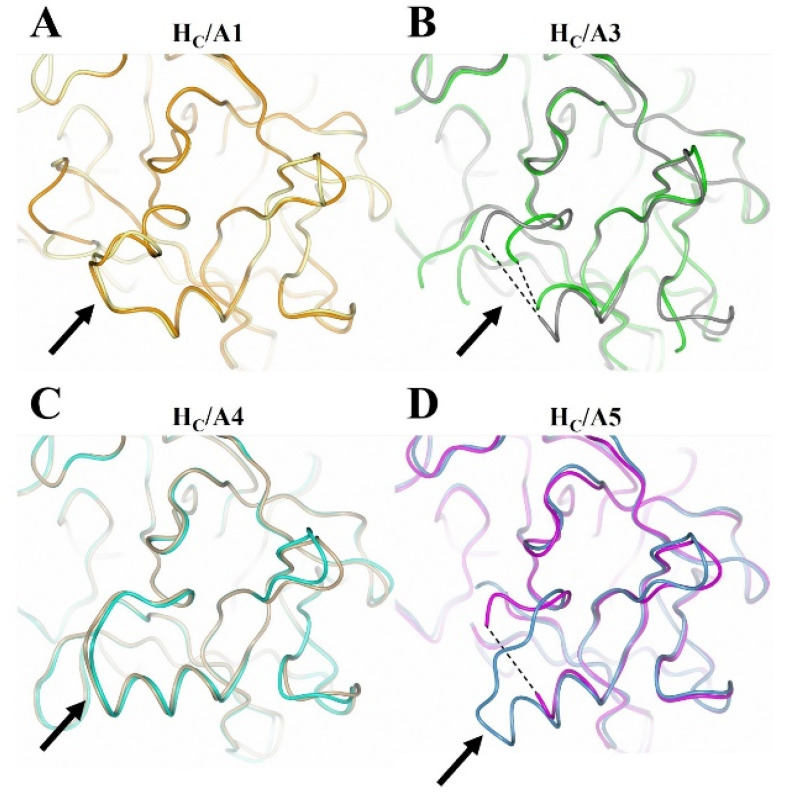
Structural comparison of the loop that follows the β-hairpin of the ganglioside binding site with and without oligosaccharide. (**A**) H_C_/A1 unbound (yellow; PDB: 2VUA [29]) and bound to GD1a (orange; PDB: 5TPC [22]); (**B**) H_C_/A3 unbound (green; PDB: 6F0O [24]) and bound to GD1a (dark grey; PDB: 6THY [23]); (**C**) H_C_/A4 unbound (burlywood; PDB: 6F0P [24]) and bound to GD1a (cyan; this study); (**D**) H_C_/A5 unbound (grey; PDB: 6TWP [25]) and bound to GM1b (cyan; this study). The arrow points to the loop that follows the GBS-β hairpin; dotted lines indicate unmodeled regions of the loop.

**Figure 7:**
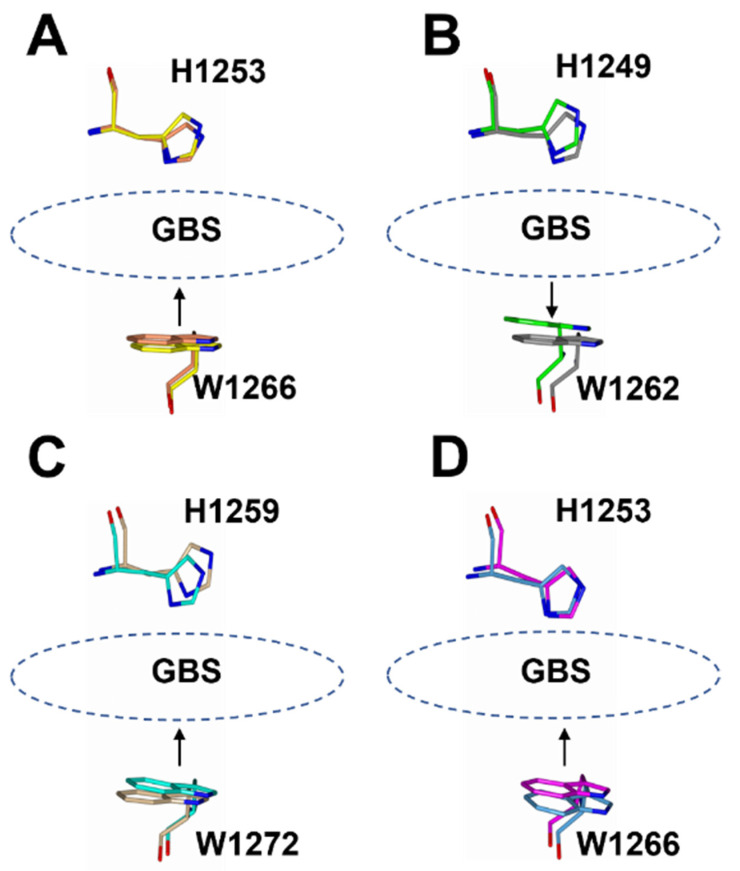
Superimposition of the opening groove between His and Trp residues of the GBS for ganglioside bound and unbound H_C_/A subtypes. For H_C_/A1 (**A**), H_C_/A4 (**C**), and H_C_/A5 (**D**), the Trp residue moves towards the His residue, whereas for H_C_/A3 (**B**) it moves away.

There appears to be some structural variation of the GBS among the H_C_/A subtypes as illustrated by a comparison of the H_C_/A1, H_C_/A3, H_C_/A4, and H_C_/A5 structures with and without ganglioside bound (Figure 5). The most significant variation is seen within the loop that follows the β-hairpin of the GBS for H_C_/A3 and H_C_/A5 (Figure 6A–D, Arrow). Upon binding the ganglioside, the loop widens in H_C_/A3 and H_C_/A5, as measured by an increase in the distance between Cα atoms of T1273^A3^/1277^A5^ and Y1263^A3^/1267^A5^ within the loop, to accommodate the ganglioside. In contrast, the loop in the unbound H_C_/A1 and H_C_/A4 structures, adopts a more open conformation, which suggests that it does not need to move to allow GD1a to bind. Furthermore, a comparison of the GBS opening groove, formed by the histidine and tryptophan residues of the H…SxWY motif, in the bound and unbound structures reveals that the structural changes of H_C_/A4 is more similar to H_C_/A1 than H_C_/A3, with the tryptophan moving towards the GBS upon ganglioside binding (Figure 7A–D). H_C_/A5 is somewhere in between with some conformational variation reminiscent of the H_C_/A3 structure, where Phe 1274^A3^/1278^A5^ appears to flip towards the GBS upon binding. This residue is not conserved across the subtypes—it appears as Leu 1278 for H_C_/A1 and Leu 1285 for H_C_/A4. Not surprisingly, there is some variation to the ganglioside interaction between subtypes. H_C_/A1 has a total of ten hydrogen bonding interactions with GD1a, while H_C_/A3 and /A4 has nine (Table 2). Furthermore, H_C_/A4 displays no water-mediated interactions with the ganglioside, while both H_C_/A1 and H_C_/A3 have at least two each. The occupancy of Gal^4^ and Sia^5^ in the H_C_/A5:GM1b structure was too low to be able to determine any water-mediated interactions that contributed to binding.

## 3. Conclusions

The crystal structures of H_C_/A4:GD1a and H_C_/A5:GM1b presented here reveal the interactions involved with ganglioside binding and also the conformational changes that occur. For H_C_/A4, eight residues form a total of nine hydrogen bonding interactions with the three principal oligosaccharides, GalNAC^3^, Gal^4^, and Sia^5^. However, for H_C_/A5, only two oligosaccharides could be modelled in the electron density map, revealing seven hydrogen bonding interactions. The low occupancy of GM1b, and multiple failed attempts of co-crystallising H_C_/A5 with GD1a, suggested a low affinity to the Sia-Gal-GalNAc moiety or preference for a different ganglioside.

A total of four H_C_/A subtype structures (H_C_/A1, H_C_/A3, H_C_/A4, H_C_/A5) have now been reported with and without ganglioside. We previously reported that the reduction in hydrogen bonding interactions of H_C_/A3 for GD1a compared to H_C_/A1, may be a contributing factor in its reduction in toxicity [23]. H_C_/A4 follows this trend as the structure displays a reduction in hydrogen bonding interactions with GD1a and has a reported 1000-fold lower activity in mice [30]. Furthermore, both BoNT/A3 and BoNT/A4 are significantly less active in vivo when compared to BoNT/A1, and BoNT/A4 is also less efficient at entering cells [31], with the cell binding domain contributing to this variation.

## 4. Materials and Methods

All reagents were purchased from Sigma-Aldrich (St. Loius, MO, USA), Fischer Scientific (Loughborough, UK), and Molecular Dimensions (Newmarket, UK) unless otherwise stated. GD1a and GM1b oligosaccharides were supplied by Elicityl (Crolles, France).

### 4.1. Protein Expression and Purification

The sequences of BoNT/A4 residues 870-1296 (HC/A4) and BoNT/A5 residues 871-1296 (HC/A5) were cloned into the pJ401 vector with an N-terminal hexa-histidine tag, as described previously [24,25]. Both constructs were transformed into *E. coli* strain BL21 and grown at 37 °C to an OD600 of 0.5 before induction with 1 mM IPTG for 16 h at 16 °C. Cells were harvested by centrifugation. Cells expressing H_C_/A4 were lysed in 50 mM Tris pH 7.4, 0.2 M NaCl, 10 mM trehalose and 20 mM imidazole, while cells expressing H_C_/A5 were lysed in 50 mM Tris pH 7.4, 0.5 M NaCl, and 20 mM imidazole. Both proteins were captured on a GE HisTrap column and further purified by gel filtration using a GE superdex 200 column. For H_C_/A4, the running buffer was 50 mM Tris pH 7.4, 150 mM NaCl, and 10 mM trehalose, while for H_C_/A5 it was 50 mM Tris pH 7.4 and 150 mM NaCl. Both proteins were concentrated to 1 mg/mL using a 10 kDa MWCO centrifugal concentrator and flash frozen in liquid nitrogen for storage at −20 °C until required for crystallisation.

### 4.2. Protein Crystallisation

H_C_/A4 and H_C_/A5 proteins were concentrated to 5 mg/mL and the former incubated with 5 mM GD1a oligosaccharide and the latter with 5 mM GM1b oligosaccharide for 1 h at room temperature. Crystallisation screens were setup using the sitting drop vapour diffusion method in 96-3 well intelli-plates (SWISSCI, High Wycombe, UK) with a number of high throughput crystallisation conditions (Molecular Dimensions). Both a 1:1 and 2:1 protein to reservoir ratios were screened in each case. H_C_/A4 crystals grew in 0.2 M NaAcO∙3H_2_O, 20% *w/v* PEG 3350 (1:1 ratio, protein:reservoir). H_C_/A5 crystals grew in 150 mM Li_2_SO_4_, 50 mM MgCl_2_∙6H_2_O, 0.1 M HEPES pH 7.8, 4.7% *w/v* PEG 8K, 4.7% PEG 10K and 4.7% PEG 8K (1:1 ratio, protein:reservoir). Crystals were mounted directly onto a cryo-loop and flash frozen for storage in liquid nitrogen.

### 4.3. X-ray Diffraction Data Collection and Structure Determination

Diffraction images were collected at a wavelength of 0.9785 Å with 0.1° oscillation and 0.01 s of exposure time per image on the i04 beamline at the Diamond Light Source (Harwell, Oxfordshire, UK). Crystals were kept under a jet stream of liquid nitrogen at 100 K during data collection. A total of 7200 images were collected for H_C_/A4:GD1a, and 3600 images for H_C_/A5:GM1b. Data processing was carried out in DIALS [32] and both structures were solved by molecular replacement in PHASER [33] using a previously reported structure of H_C_/A4 [24] and H_C_/A5 [25] as search models. Initial rounds of refinement were performed using REFMAC [26] as part of the CCP4 package [34] with the final round of refinement and validation performed in Phenix [35]. The structures were validated using Molprobity [36] and PDB validation. Figures were produced using ccp4mg [37] and BioRender.com (Biorender, Toronto, ON, Canada).

## Figures and Tables

**Table 1:** X-ray crystallographic data collection and refinement statistics for H_C_/A4:GD1a and H_C_/A5:GM1b. Outer shell statistics are in parentheses.

**Beamline**	I04	
**Wavelength used**	0.9795 Å	
**Protein**	**H_C_/A4:GD1a**	**Hc/A5:GM1b**
**Crystallographic Statistics**		
Space group	P6_1_	P2_1_
Unit cell dimensions:		
a, b, c (Å)	94.68, 94.68, 181.21	44.16, 129.40, 78.05
α, β, γ (°)	90.00, 90.00, 120.00	90.00, 102.96, 90.00
Resolution range (Å)	90.60–2.30 (2.38–2.30)	129.40–2.30 (2.49–2.40)
*R*_merge_	0.269 (1.866)	0.305 (1.586)
*R_pim_*	0.043 (0.366)	0.126 (0.746)
*<I/σ(I)>*	12.1 (1.9)	4.3 (0.8)
*CC*_1/2_	0.998 (0.644)	0.982 (0.575)
Completeness (%)	99.7 (97.5)	100.0 (100.0)
No. observed reflections	1,594,764 (97,629)	227,781 (22,543)
No. unique reflections	40,672 (3862)	33,451 (3528)
Multiplicity	39.2 (25.3)	6.8 (6.4)
**Refinement Statistics**		
*R*_work_/*R*_free_	0.203/0.248	0.222/0.262
RMSD bond lengths (Å)	0.004	0.003
RMSD bond angles (°)	0.68	0.55
Ramachandran plot statistics (%):		
Favoured	95.44	95.38
Allowed	4.56	4.34
Outliers	0.00	1.02
Average B-Factors (Å^2^):		
Protein atoms	42.28	39.10
Solvent atoms	35.04	32.36
GD1a atoms	72.59	70.99
No. Atoms:	7069	6286
Protein	6696	6094
Solvent	237	160
GD1a/GM1b carbohydrates	136	32
PDB code	7QPT	7QPU

**Table 2:** Hydrogen bonds for ganglioside binding in structures H_C_/A4:GD1a and H_C_/A5:GM1b (this study), compared to H_C_/A3:GD1a (PDB: 6THY [23]) and H_C_/A1:GD1a (PDB: 5TPC [22]). Water-mediated interactions are indicated by a “-H_2_O molecule (n_1_, n_2_)“ where n_1_ is the distance between the amino acid residue and the water, and n_2_ is the distance between the water and monosaccharide. ^Δ^ Indicates they are the equivalent water molecule for each structure. Data adapted from [23].

Monosaccharide	H-Bonding Residue (Distance in Å)			
	H_C_/A5:GM1b	H_C_/A4:GD1a	H_C_/A3:GD1a	H_C_/A1:GD1a
Sia^6^	N/A	Not modelled	Not modelled	Trp 1266 (3.5)
Sia^5^	Tyr 1117 (2.8)	Tyr 1123 (2.8)		Tyr 1117 (2.9)
	Tyr 1267 (2.7)	Tyr 1273 (2.5)	Tyr 1263 (2.7)	Tyr 1267-H_2_O (2.5, 3.5)
	Gly 1279 (3.2)	Gly 1285 (3.1)	Gly 1275 (2.9)	Gly 1279-H_2_O ^Δ^ (2.6 2.8)
			Leu 1250-H_2_O ^Δ^ (2.9, 2.8)	
		Arg 1282 (3.8)		Arg 1276 -H_2_O ^Δ^ (2.8, 2.8)
Gal^4^	Glu 1203 (2.6)	Glu 1209 (2.4)	Glu 1199 (2.7)	Glu 1203 (2.8)
	Phe 1252 (2.8)	Phe 1258 (2.8)	Phe 1248 (2.5)	Phe 1252 (2.7)
	His 1253 (3.1)	His 1259 (2.7)	His 1249 (3.1)	His 1253 (2.7)
	Ser 1264 (2.9)	Ser 1270 (2.5)	Ser 1260 (2.7)	Ser 1264 (2.8)
			Leu 1250-H_2_O ^Δ^ (2.9, 3.0)	
GalNAc^3^	Not modelled	Glu 1209 (2.6)	Glu 1199 (2.5)	Glu 1203 (2.5)

## Data Availability

The atomic coordinates and structure factors of H_C_/A4-GD1a and H_C_/A5-GM1b have been deposited in the protein data bank under the accession codes 7QPT and 7QPU, respectively.
